# Wnt signaling and orthopedics, an overview

**DOI:** 10.3109/17453674.2011.572252

**Published:** 2011-04-05

**Authors:** Fredrik Agholme, Per Aspenberg

**Affiliations:** Orthopedics, Department of Clinical and Experimental Medicine, IKE, Faculty of Health Sciences, Linköping University, Linköping, Sweden

## Abstract

Wnt signaling is a ubiquitous system for intercellular communication, with multiple functions during development and in homeostasis of the body. It comprises several ligands, receptors, and inhibitors. Some molecules, such as sclerostin, appear to have bone-specific functions, and can be targeted by potential drugs. Now, ongoing clinical trials are testing these drugs as treatments for osteoporosis. Animal studies have also suggested that these drugs can accelerate fracture healing and implant fixation. This brief overview focuses on currently available information on the effects of manipulations of Wnt signaling on bone healing.

**Abbreviations**
BMP: Bone morphogenic protein
Dkk: Dickkopf
Frzb: Frizzled-related protein
Fz: Frizzled
GSK3ß: Glycogen synthase kinase-3 ß
Lef: Lymphoid enhancer binding factor
LRP4, -5, -6: Low-density lipoprotein receptor-related proteins 4, 5, and 6
OPG: Osteoprotegerin
PTH: Parathyroid hormone
RANKL: Receptor activator of nuclear factor kappa B ligand
sFrp: Secreted Frizzled-related protein
Tcf: T-cell factor
TNF-α: Tumor necrosis factor α
Wif1,2: Wnt inhibitory factor
Wise: Wnt modulator in surface ectoderm
Wnt2, -3a, -4, -5a, -5b, -10: Wnt ligand 2, 3a, 4, 5a, 5b, and 10
Wnts: Wnt ligands

## What is Wnt signaling?

When the orthopedics community learned about bone morphogenetic proteins (BMPs) in the 1990s, the expectations about new therapeutic possibilities may have been unrealistic, but after a long delay some of these expectations have actually been met (Agarwal et al. 2009). The BMPs are part of a ubiquitous signaling system with some specific functions in bone. They are not alone, however: another signaling system has recently turned out to be important for bone homeostasis and regeneration, with perhaps even greater potential for therapeutic application—namely Wnt signaling. Drugs that interfere with this pathway are now close to clinical testing for acceleration of fracture healing. Similarly to PTH, these drugs might become useful tools for the orthopedic surgeon.

This paper is not a formal literature review, but is intended to give an overview of studies of Wnt signaling that are of relevance to the field of orthopedics. We searched Pubmed and clinicaltrials.gov with the terms “Wnt bone formation”, “Wnt bone fracture”, “Wnt osteoarthritis”, and “Wnt bone implant”. Review articles and original work were included. We excluded studies that focus on cancer and those with only in vitro data, and tried to make a synthesis of the 174 articles that remained.

### Wnts are secreted signaling proteins that increase intracellular ß-catenin

Natural mutations in humans gave the first indication of the importance of Wnt signaling in bone formation. The affected subjects had a several-fold increase in bone mass, with few other changes. Study of these mutations in transgenic animals suggested a therapeutic potential for drugs that interfere with Wnt signaling to increase bone mass.

Wnt ligands (Wnts) are a group of secreted proteins that are important for embryonic development, as well as cell proliferation and differentiation in the adult (Logan and Nusse 2004). The complete signaling process has been reviewed in detail by others (Logan and Nusse 2004, MacDonald et al. 2009, Macsai et al. 2008), and we will only describe it briefly before discussing its possible importance for orthopedics. Currently, 19 Wnt homologs have been described in humans, with a wide range of functions and expression patterns. The name Wnt is derived from a combination of Wg (Wingless gene in Drosophila) and Int-1 (gene from the integration site of mouse mammary tumor virus). It was coined when these two genes were shown to be homologous (Rijsewijk et al. 1987).

**Figure 1.**
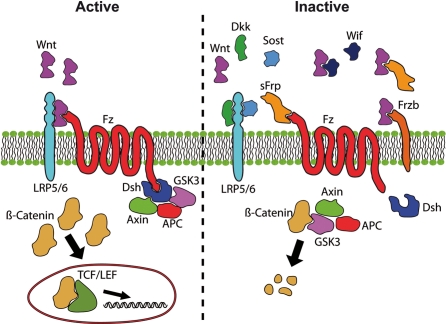
Canonical Wnt signaling. In the active state, Wnt ligands (Wnt) form a complex with the receptors low-density lipoprotein receptor-related protein 5 or 6 (LRP5/6) and Frizzled (Fz). Disheveled (Dsh) is then able to bind to Fz. Dsh forms a complex with glycogen synthase kinase 3ß (GSK3ß), Axin, and adenomatous polyposis coli (APC). This protects ß-catenin from proteasomal degradation so that ß-Catenin can accumulate in the cytosol and translocate to the nucleus. In the nucleus it interacts with the T-cell factor/lymphoid enhancer factor (TCF/LEF) family of transcription factors, leading to gene transcription. Inhibitors of this system prevent the formation of the Wnt-Fz-LRP5/6 complex, inactivating Wnt signaling. This leads to GSK3ß-mediated phosphorylation of ß-catenin, causing it to be degraded. A variety of extracellular inhibitors serve as inactivators. Dickkopf (Dkk) and sclerostin (Sost) bind to LRP5/6, preventing Wnt binding. Secreted Frizzled-related protein (sFrp) has similarities with Frizzled, and can bind either to the Wnt ligand or to the Fz receptor itself. Frizzled-related protein (Frzb) acts as a decoy receptor for Wnt ligands. Wnt-inhibitory factor (Wif) also binds directly to the Wnt ligand (Komatsu and Warden 2010, MacDonald 2009, Macsai 2008).

Wnts interact with receptors that activate several sets of intracellular signaling pathways. These pathways can be subdivided into canonical Wnt signaling and non-canonical Wnt signaling. Canonical Wnt signaling is the most studied, and this overview will center on this pathway, since it appears to be the most important in bone. The hallmark of canonical Wnt signaling is the stabilization of ß-catenin in the cytosol, which enables it to translocate to the nucleus and regulate gene expression (Figure 1). In contrast, the non-canonical pathways function without ß-catenin. Initially, Wnts bind to a specific receptor belonging to the Frizzled (Fz) group (there are at least 10 of them). A receptor complex is then formed with low-density lipoprotein receptor-related proteins (LRPs) 4, 5, and 6. This event prevents an intracellular protein complex consisting of Axin, GSK3ß, and APC from tagging ß-catenin for degradation. As a result, ß-catenin accumulates in the cytosol and can translocate into the nucleus, where it interacts with members of the Tcf/Lef class of DNA binding proteins and transcriptions factors. Precise regulation of this system is vital, especially in embryonic development. Correct formation and function of the nervous system, brain, heart, and kidneys is also dependent on this system (Macsai 2008). Wnt signaling is also implicated in cancer, by increasing cell proliferation (MacDonald 2009). Far from being isolated, Wnt signaling is prone to crosstalk with other pathways, notably those connected to PTH (Guo et al. 2010) and BMPs (Itasaki and Hoppler 2010).

### Wnt signaling is regulated by soluble inhibitors

There are several feedback loops, with both secreted and intracellular inhibitors, which modify Wnt signaling (Figure 1). These secreted inhibitors include sclerostin (the product of the SOST gene), the Dickkopfs (Dkks), secreted Frizzled-related proteins (sFrps), Frizzled-related protein (Frzb), Wnt1-induced secreted protein (WISE), Wnt inhibitory factor-1 and -2 (Wif-1, Wif-2) and Chibby (Galli et al. 2010). The secreted inhibitors are particularly interesting, as they can be targeted by therapeutic antibodies, some of which appear to be efficacious and safe in clinical settings.

## Wnt signaling in development

### Wnt signaling is required to establish the head–to-tail axis

In all animals studied, Wnt signaling is crucial for embryonic development (van Amerongen and Nusse 2009). It is required for processes that regulate the establishment of head–to-tail axis, limb polarity, neural crest differentiation, kidney morphogenesis, and sex determination. Disruption of this pathway can lead to major developmental disabilities. It is not only the Wnt ligands that need to be present; Wnt inhibitors are equally important. For example, embryos lacking Dickkopf-1 (Dkk1) do not develop heads (hence the name Dickkopf) (Glinka et al. 1998). In contrast, when the inhibitors sclerostin or sFrp1 are absent in vivo, the only tissue that appears to be affected is the skeleton (Li et al. 2008, Gaur et al. 2009). The receptors LRP5 and LRP6 have received much attention, since they seem to be partially redundant but still vital to development. In vivo experiments have shown LRP6-deficency to be fatal, while animals with a deficiency in LRP4 and LRP5 are viable (MacDonald et al. 2009). These developmental studies have been helpful in elucidating the important role of Wnt signaling in cell proliferation and differentiation—not just in bone, but also in other tissues.

## Wnt signaling in bone

### Wnt signaling is important for bone maintenance and repair

ß-catenin is needed both to promote early osteoblast proliferation and differentiation (Galli et al. 2010) and to suppress osteoclasts. ß-catenin accumulation favors mesenchymal stem cell commitment for an osteogenic fate, away from the adipogenic or chondrogenic lineage (Case and Rubin 2010). Precise regulation of ß-catenin via Wnt signaling is needed for a proper healing response (Chen et al. 2007, Kim et al. 2007), and mutations in parts of this system either lead to excessive bone growth or to bone resorption. For example, loss-of-function mutations in LRP5 lead to the human osteoporosis pseudoglioma syndrome, with low bone mass (Gong et al. 2001), whereas gain of function leads to high bone mass (Boyden et al. 2002, Little et al. 2002, Semenov and He 2006). Consequently, loss-of-function mutations affecting the inhibitor sclerostin (van Bezooijen et al. 2005, Semenov and He 2006) give rise to a very high bone mass phenotype in humans, characterized by generalized cortical hyperostosis—van Buchem's disease (Balemans et al. 2002).

### Wnt signaling is involved in the response of bone to mechanical loading

**Figure 2.**
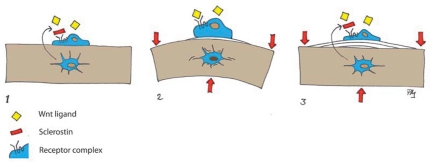
Wnt signaling in mechanotransduction: 1. The Wnt inhibitor sclerostin is secreted from osteocytes and blocks stimulation of lining cells. 2. Deformation due to mechanical loading is perceived by osteocytes, which reduce sclerostin secretion, thus permitting surface cells to be activated by Wnt ligands. 3. Bone apposition reduces deformation and sclerostin secretion is increased again.

Sclerostin also plays an important role in how bone responds to mechanical loading (Lin et al. 2009, Robling et al. 2008) (Figure 2). There are also some indications that Dkk1 is involved, although to a lesser degree than sclerostin, in the response of bone to mechanical loading (Robling et al. 2008). Furthermore, deficiency of one or both of the genes for the inhibitor Dkk1 leads to increased bone mass and stronger bone (MacDonald et al. 2007, McDonald et al. 2010, Morvan et al. 2006). Lithium is an inhibitor of GSK3ß, which is a protein in the intracellular cascade that phosphorylates ß-catenin. Lithium thus increases ß-catenin levels, which promotes bone formation and increases bone mass in mice (Clement-Lacroix et al. 2005). There are some indications that it may also have these effects in the clinic (Vestergaard et al. 2005, Zamani et al. 2009).

Some common polymorphisms of the Wnt receptor LRP5 gene are associated with osteoporotic fractures, and polymorphisms of LRP6 are associated with low bone mineral density, thus explaining some of the influence of heredity on osteoporosis (Riancho et al. 2010). These mutations point to the possibility of modulating Wnt signaling for orthopedic purposes, as few organs except bone appear to be affected. Moreover, these effects arise after lifelong exposure, suggesting that limited short-term use of agents that modulate the pathway could be safe and suitable for clinical practice.

## Wnt signaling in fracture healing

### Increased Wnt signaling improves bone healing

ß-Catenin is important for bone healing (Chen et al. 2007, Kim et al. 2007) and consequently, modulation of Wnt signaling has been shown to influence fracture healing. The Table is a summary of the modulation methods used to enhance healing. In an endochondral setting, ß-catenin appears to have different effects at different stages of bone repair. Early in the process, it controls the relation between the numbers of osteoblasts and chondrocytes that arise from the pluripotent mesenchymal cells. Thus, either too much or too little ß-catenin can be detrimental to bone healing at this stage. Later on, ß-catenin promotes the differentiation of osteoblasts and enhances their production of bone matrix, so that too little ß-catenin at this stage impairs healing whereas raised ß-catenin levels improve healing (Chen et al. 2007). Gene expression studies in diaphyseal fractures in rats have shown increased ß-catenin expression on day 3 after fracture, peaking at 10 days and leveling out at 21 days, but remaining up-regulated thereafter (Hadjiargyrou et al. 2002, Zhong et al. 2006). Another study in mice showed that the ligands Wnt4, 5a, 5b, 10b, and also Dkk1 and sclerostin were upregulated in a similar pattern, with a peak around day 10 but quite low expression during the first days. On the other hand, the receptors LRP5 and LRP6 were upregulated from day 1 (Kakar et al. 2007). Moreover, in the same study, PTH treatment increased the expression of these Wnt factors, indicating that there was interaction between PTH and Wnt signaling (Kakar et al. 2007). This connection may be important, because intermittent PTH injections are the only systemic treatment that has so far been shown to improve fracture healing clinically (Aspenberg et al. 2010, Aspenberg and Johansson 2010).

**Table:** Changes in Wnt signaling and fracture healing

Component	Role	Administration	Animal	Fracture model	Main finding	Reference
Wnt3a	Ligand	Liposomal, local	Mouse	Proximal tibia	Enhanced bone regeneration by Wnt3a administration	Minear et al. 2010
Sclerostin	Inhibitor	Antibody, systemic	Rat	Proximal tibia	Increased bone formation in both traumatized and untraumatized bone	Agholme et al. 2010
Sclerostin	Inhibitor	Antibody, systemic	Rat / Monkey	Femur shaft / fibular osteotomy otomy	Reduced cartilage formation and improved fracture healing	Ominsky et al. 2010
Dkk1	Inhibitor	Antibody, systemic	LRP5-/- Mouse	Femur shaft	Improved fracture healing	Komatsu et al. 2010
Dkk1	Inhibitor	Antibody, systemic	Rat	Proximal tibia	Increased bone formation and improved implant fixation and fracture repair	Agholme et al. 2011
sFrp1	Inhibitor	Genetic modification	sFrp1-/- Mouse	Tibia shaft	Faster and better fracture repair	Gaur et al. 2009
LiCl	Inhibitor	Oral	Mouse	Tibia shaft	Treatment improves fracture healing if initiated after the fracture has occurred	Chen et al. 2007

### Wnt signaling stimulates direct bone formation

Wnt signaling is also important for metaplastic (intramembranous) bone formation, as has been shown in bone healing models without cartilage formation. The healing of drill holes in the mouse proximal tibia is dependent on Wnt-mediated ß-catenin signaling (Kim et al. 2007). Gene expression during intramembranous bone formation caused by marrow ablation has been studied in rats at several time points after the injury. Genes involved in Wnt signaling were found to be upregulated, with a peak after 10 days and then leveling out. Ligands were upregulated (Wnt2, -5a, and -5b) as well as receptors (LRP4 and -6, and several of the Fz receptors) and inhibitors (sFrps, Wise, and Frzb) (Wise et al. 2010).

### Blocking of either of the Wnt inhibitors sclerostin and Dkk-1 alleviates osteoporosis and stimulates fracture healing

There is firm evidence for the importance of Wnt signaling in bone healing. Thus, there have been efforts to influence healing by blocking the inhibitors of the Wnt pathway. Two inhibitors in particular have been studied more extensively, namely sclerostin and Dkk1. Both sclerostin and Dkk1 inhibit only canonical Wnt signaling and their main effects appear to be on the skeleton. Antibodies that block sclerostin are therefore being evaluated for osteoporosis treatment. They increase bone mass and counter the effects of ovariectomy in both rodents and monkeys (Li et al. 2009, 2010, Ominsky et al. 2010). A phase-2 clinical trial has indicated that they are safe and lead to increasing bone density in osteoporosis (Padhi et al. 2011). In addition, clinical trials regarding fracture healing are under way.

Slerostin-blocking antibodies improve screw fixation and increase intramembranous bone formation in the proximal tibia of rats (Agholme et al. 2010), and also in midshaft fractures in rats (Ominsky et al. 2010). These antibodies also counter the effects of mechanical load deprivation, highlighting the importance of sclerostin as a mechanotransducer in bone (Tian et al. 2010). In a similar way, in mice, antibodies to Dkk1 increase bone volume and density (Glantschnig et al. 2010), increase callus size and bone formation (Komatsu et al. 2010), and protect against inflammatory bone loss (Diarra et al. 2007). Furthermore, data from our laboratory suggest that inhibition of Dkk1 with antibodies has effects on implant fixation and bone regeneration that are similar to those of anti-sclerostin antibodies (Agholme et al. 2011). Both types of antibodies attenuated bone loss in the proximal rat tibia after mechanical unloading, However, neither of them alone was able to completely preserve bone mass, suggesting redundancy in this signaling system (own unpublished data).

### There may be more ways to increase Wnt signaling

Knockout mice lacking sFrp1 have higher bone mass and diaphyseal fractures heal more quickly. The faster healing, with no loss of bone quality, is due to increased metaplastic, direct bone formation and less cartilaginous callus (Gaur et al. 2009). Increased osteoclast activity was also noted, but this could be attributed to the need for more woven bone to be remodeled.

The intracellular pathway of canonical Wnt signaling, i.e. the regulation of ß-catenin levels, appears to also disclose targets for drug treatment. Thus, inhibition of GSK3ß using lithium has a positive effect on bone formation if administered after the initiation of trauma (Chen et al. 2007). However, unpublished data from our laboratory, using oral administration of lithium, have not shown any substantial effects on bone healing like the ones experienced with antibodies to sclerostin or Dkk1. Other drugs can also modify GSK3ß function and promote the differentiation of osteogenic progenitors (Gambardella et al. 2010). However, GSK3ß appears to have many important functions apart from regulation of bone, and is also regulated by systems other than Wnt signaling. Thus, drugs that target ß-catenin directly may have too many adverse effects.

Another way of influencing Wnt signaling is to supply more Wnt ligand. Wnt3a, applied locally to a drill hole in the proximal tibia of mice, induced upregulation of ß-catenin expression and accelerated bone healing (Minear et al. 2010). Local administration of Wnt3a increased peri-implant bone formation around stainless-steel implants in mice. This transient delivery of Wnt ligand increased the differentiation of peri-implant cells towards an osteoblastic phenotype (Popelut et al. 2010). The importance of this for implant osseointegration is also supported by another study, in which both Dkk1 and Dkk2 were found to influence early osteoblast differentiation on titanium surfaces in vitro. The expression of these genes was also dependent on implant surface specifications (Olivares-Navarrete et al. 2010). In summary, increased Wnt signaling seems to enhance bone formation, and this can be achieved either by administration of more Wnt ligand or by removal of an inhibitor.

## Wnt signaling and joint disease

### Dkk1 is involved in joint destruction and osteophyte formation

Dkk1 appears to be a key regulator of pathological bone remodeling in joint disease. Changes in Dkk1 expression may be responsible for many of the differences in radiological appearance between osteoarthritis and rheumatoid arthritis. Overexpression of TNF-α in transgenic mice leads to synovitis and joint destruction similar to that in rheumatoid arthritis. TNF-α also increases Dkk1 expression in the inflamed synovium, leading to inability to repair the arthritic erosions. When Dkk1 was blocked with an antibody, no joint destruction developed, in spite of the TNF-α. Instead, osteophytes formed, making the joint appear osteoarthritic (Diarra et al. 2007). Dkk1 was induced by TNF-α but did not participate in inflammation: it had its sole effect on bone. Dkk1 can also increase the expression of sclerostin, thus further exacerbating the inability to replace bone that has been lost due to inflammation (Heiland et al. 2010). In contrast to the upregulation with inflammation induced by TNF-α, Dkk1 is downregulated in ankylosing spondylitis and osteoarthritis. This leads to increased bone formation and the formation of osteophytes (Diarra et al. 2007). Downregulation of Dkk1 is possibly responsible for the ankylosis in spondylitis. There are also complex cross reactions with the regulation of osteoclast activity via the OPG /RANKL system.

## What's in it for the orthopedic surgeon?

### Wnt signal modulators may become orthopedic tools

We believe that in the future, orthopedic surgeons and rheumatologists will use drugs that the modulate the Wnt signaling system. Especially in fracture treatment, the use of inhibitory antibodies is likely to be safe and cost-effective—considering the limited treatment time. Because some of the Wnt inhibitors are expressed and secreted only in bone, antibodies to them will have few side effects outside the skeleton. The orthopedic toolbox will contain several drugs with some effects on bone healing, such as BMPs, PTH, and Wnt modulators. However, their effects will be different. It is now clear that BMP-2 performs better than cancellous autografts for spine fusion (Agarwal et al. 2009). BMPs are known for their ability to induce bone formation from unconditioned cells, mainly via the endochondral pathway. This is also the cause of some of its adverse effects. In contrast, sclerostin antibodies can only function if sclerostin-producing bone cells are already at hand, and will then favor metaplastic bone formation. PTH appears to lie somewhere between BMPs and sclerostin antibodies, in that it mainly stimulates cells that already belong to the osteoblastic lineage, but it does not require the mature cells that produce sclerostin. However, its efficacy may be limited because of dosage problems. In contrast to BMPs, PTH and sclerostin antibodies must be given systemically and do not require surgery. The future will tell about the results of ongoing human fracture trials with sclerostin antibodies.
